# Draft Genome Sequence of the Vaccination Strain *Mycobacterium bovis* BCG S4-Jena

**DOI:** 10.1128/genomeA.00296-16

**Published:** 2016-04-21

**Authors:** Daniel Wibberg, Anika Winkler, Eberhard Straube, Matthias Karrasch, Peter M. Keller, Jörn Kalinowski

**Affiliations:** a Technology Platform Genomics, Center for Biotechnology (CeBiTec), Bielefeld University, Bielefeld, Germany; b Institute of Medical Microbiology, Jena University Hospital, Friedrich-Schiller University, Jena, Germany

## Abstract

Here, we present the draft genome sequence of *Mycobacterium bovis* BCG S4-Jena, a tuberculosis vaccine strain. The genome of S4-Jena is represented by 48 scaffolds, consisting of 132 scaffolded contigs and amounting to a size of about 4.2 Mb. New genes potentially encoding a phage fragment were identified in the genome.

## GENOME ANNOUNCEMENT

From its initial release at the Pasteur Institute in Paris in 1921, as an attenuated live vaccine for tuberculosis, until the introduction of lyophilized seed lots in the 1960s, *Mycobacterium bovis* Bacille Calmette-Guérin (BCG) was distributed worldwide and cultivated by continuous serial passage (1). As a result, compared with the original 1921 strain (substrain Pasteur; not available anymore), cultivated BCG strains accumulated mutations such as single-nucleotide polymorphisms and genetic loss of regions of difference (2, 3).

The tuberculosis vaccine strain S4-Jena (strain accession DSM 45071) was brought to Jena, Germany, from Gothenburg, Sweden, in 1950 by the microbiologist Hans Knöll (4). Allegedly, it is a derivative of a Swedish BCG substrain that was imported directly from the Paris Pasteur Institute in the 1920s. From 1950 onward, it was used to prepare tuberculosis vaccine batches to be used in the former German Democratic Republic (GDR) and in Poland. As such, S4-Jena showed no significant side effects such as systemic infections in neonates (5). The S4-Jena strain was examined in a phase II clinical multicenter trial as adjuvant therapy of nonmuscle invasive bladder cancer in 7 hospitals in the former GDR from 1988 to 1991. It was shown that 80.2% of the patients had no recurrence within the follow-up period (16.4 months). Based on this study, the S4-Jena strain was licensed for bladder cancer therapy in eastern Europe in 1990 (4). With the recent knowledge that two phylogenetically different BCG substrains have different clinical outcomes, if used to treat bladder cancer (i.e., the phylogenetically older BCG Connaught strain conferred significantly greater 5-year recurrence-free survival than BCG Tice), we aimed to genetically characterize a formerly broadly used, phylogenetically old BCG strain with a known safety record (6). In comparison to the commercially available BCG substrains Tice (OncoTICE) and Connaught (immuCyst) that are licensed to treat nonmuscle invasive bladder cancer, this strain has fewer genome alterations in comparison with the wild-type *M. Bovis*.

To determine the draft genome sequence of *M. bovis* BCG S4-Jena, sequencing of a paired-end library on the Illumina MiSeq system was performed. The sequencing run (2 × 250-bp) resulted in 2,570,425 reads, yielding approximately 587 Mb of sequence information. A *de novo* assembly of the processed reads by means of gsAssembler version 2.8 software generated 382 contigs, accounting for a total length of 4.3 Mb and featuring a GC content of 65.55%. The assembly was validated by applying contig-length versus read-count analysis (7, 8). The remaining assembled contigs could be arranged in 48 scaffolds with 132 scaffolded contigs by exploiting paired-end sequence information. For gene prediction and functional annotation, the genome annotation platform GenDB (9) was applied. This approach resulted in 4,020 predicted genes. In total, 67 single-nucleotide polymorphisms were detected in comparison with the *M. bovis* BCG Pasteur 1173 strain. 

### Nucleotide sequence accession numbers.

The *M. bovis* BCG S4-Jena genome project has been deposited in the DDBJ/EMBL/GenBank database under the accession numbers CYST01000001 to CYST01000131. The version described in this paper is the first version.
